# Associations between global postural factors and mandibular lateral deviation during maximal mouth opening in healthy young adults: cross-sectional study

**DOI:** 10.1186/s12998-026-00632-4

**Published:** 2026-03-13

**Authors:** Yoongyeom Choi, Feifei Li, Ilyoung Moon, Chunghwi Yi

**Affiliations:** 1 Department of Physical Therapy, The Graduate School, Yonsei University, Wonju,, Korea; 2 Department of Physical Therpay, Hallym Sacred Heart University, Chuncheon, Korea; 3 Department of Physical Therapy, College of Health Sciences, Yonsei University, Wonju, Korea

**Keywords:** Mandibular lateral deviation, Global posture, Temporomandibular joint, Maximal mouth opening, Lower extremity

## Abstract

**Background:**

Mandibular lateral deviation during mouth opening may reflect temporomandibular joint and masticatory dysfunction. While postural factors are implicated in temporomandibular joint disorders, the relationship between global posture and mandibular lateral deviation in healthy individuals remains unclear. Therefore, this study aimed to investigate the correlations and associations between mandibular lateral deviation during maximal mouth opening and global postural factors in healthy young adults.

**Methods:**

This cross-sectional study involved 110 healthy adults (aged 20–39 years) recruited between September 2024 and August 2025 through advertisements at Yonsei University and in downtown Wonju, Gangwon, South Korea. Twelve postural variables were assessed using a three-dimensional posture analysis system: head posture, shoulder height difference, rounded shoulder, thoracic kyphosis, lumbar lordosis, spinal lateral deviation, three pelvic parameters (pelvic tilt, rotation, obliquity), left/right hip-knee-ankle angles, and knee flexion angle. Mandibular kinematics, including lateral deviation and maximal mouth opening distance, were assessed using video-based motion analysis. Relationships with global postural parameters were evaluated using Spearman’s correlation and stepwise multiple regression analyses.

**Results:**

The regression model exhibited an adjusted *R*^2^ of 0.193, and knee flexion angle (β = − 0.415, *p* < 0.001) and spinal lateral deviation (β = − 0.180, *p* = 0.041) emerged as significant factors associated with mandibular lateral deviation.

**Conclusion:**

These findings suggest a potential interrelationship between global posture, specifically lower limb and spinal alignment, and mandibular function. However, further longitudinal and interventional studies are warranted to confirm these associations and clarify causal mechanisms.

## Background

Mandibular movement assessment is a critical indicator evaluating temporomandibular joint (TMJ) function in clinical practice [1, 2]. While TMJ movements typically involve bilateral coordination between the working and non-working sides, asymmetric motion may occur. This asymmetry is manifested as mandibular lateral deviation (MLD), particularly in the presence of muscular imbalances or articular pathology [3, 4].

The stomatognathic system is intricately linked to global posture through interconnected neuromuscular and myofascial networks supported by various connective tissues (e.g., ligament, fascia, and others) [5]. The myofascial system acts as a continuous tension network in which mechanical forces can be transmitted across distant regions. For example, tension or misalignment in the lower kinetic chain may propagate through the thoracolumbar fascia to the craniocervical region via connective tissue continuity [6, 7]. Alongside these mechanical interactions, sensory input from these distributed structures is integrated within the central nervous system to facilitate both mandibular positioning and global postural control [8]. This aligns with the “regional interdependence” theory proposed by Wainner et al., which posits that seemingly unrelated impairments in anatomically remote regions may contribute to, or be associated with, a patient’s primary complaint [9]. Consequently, dysfunction in one region can influence alignment and function in distant areas, providing a mechanistic basis for the frequently reported association between postural dysfunction and masticatory disorders.

To date, most prior studies have primarily focused on patients with confirmed TMJ disorders (TMDs), extensively documenting that craniocervical malalignment, such as forward head posture, contributes significantly to dysfunction owing to its direct biomechanical proximity [10–12]. However, this focus on symptomatic populations and proximal factors leaves critical gaps in our understanding. First, it remains unclear how global postural factors involving broader anatomical chains influence mandibular function before pathology develops. Second, few studies have specifically examined the relationship between MLD during maximal mouth opening (MMO) and global postural alignment, including the spine and pelvis, in asymptomatic individuals. Establishing these normative associations in healthy adults is crucial for distinguishing pathological adaptations from functional variations.

Therefore, the present study aimed to address these gaps in the literature. Unlike previous studies focusing primarily on patients with TMDs and craniocervical alignment, we investigated healthy young adults to establish normative biomechanical data. Furthermore, we expanded the scope of assessment to include lower-extremity and pelvic parameters using a validated 3D body-surface topography system. Finally, we analyzed MLD as a signed continuous variable rather than a categorical one to detect subtle biomechanical associations between global posture and mandibular movement. Within this integrated assessment of anatomical chains, we hypothesized that, given its direct anatomical proximity, craniocervical alignment would exhibit a more significant association with MLD than pelvic or lower extremity alignment.

## Methods

### Study design and participants

This cross-sectional study investigated the associations between global posture and mandibular kinematics. The study adhered to the STROBE guidelines and complied with the Declaration of Helsinki. The study protocol was approved by the Institutional Review Board of Yonsei University Mirae Campus (IRB No. 1041849-202407-BM-148-04).

Data collection was conducted from September 3, 2024, to August 20, 2025. A total of 110 healthy young adults were recruited through advertisements at Yonsei University and in downtown Wonju, Gangwon, South Korea. All participants provided written informed consent before participation. The target age range (20–39 years) was selected based on previous epidemiological data indicating a high prevalence of TMDs within this demographic [13].

Eligibility was determined based on strict exclusion criteria to ensure a functionally asymptomatic sample. Exclusion criteria were as follows: (1) scores on the Jaw Functional Limitation Scale-20 indicating the presence of chronic temporomandibular disorders; (2) loss of posterior teeth; (3) history of trauma to the head and neck; (4) craniofacial anomalies; (5) neurodegenerative disorders; (6) regular use of medications, such as nonsteroidal anti-inflammatory drugs, antidepressants, muscle relaxants, or anticonvulsants; (7) systemic diseases requiring regular medication; (8) history of botulinum toxin injections in the cranial or cervical muscles within the past year; and (9) communication disorders.

Loss of posterior teeth was included as an exclusion criterion because posterior occlusal support is essential for maintaining the vertical dimension of occlusion and stabilizing condylar position within the glenoid fossa [14]. Absence of posterior support can lead to mandibular instability and altered chewing patterns, potentially confounding the subtle biomechanical associations between global posture and mandibular movement [15].

#### Clinical screening using the jaw functional limitation scale-20

A rigorous preliminary screening was conducted to ensure that the sample represented a functionally healthy and asymptomatic population. While established diagnostic tools, such as the Research Diagnostic Criteria for Temporomandibular Disorders, are primarily designed for clinical classification of the disorder, this study utilized the Jaw Functional Limitation Scale-20 as a primary screening tool, owing to its validated capacity to effectively differentiate between individuals with no lifetime history of the disorder and those with chronic symptoms [16, 17].

The Jaw Functional Limitation Scale-20 is a comprehensive, multidimensional instrument that assesses functional limitations across three main domains: mastication, mandibular mobility, and verbal/emotional expression [18]. The Korean version of this scale has demonstrated high internal consistency (Cronbach’s α = 0.90) and reliability [19]. Based on established validation criteria, participants were excluded if they met any of the following thresholds: a global score ≥ 1.63, a mastication limitation score ≥ 2.09, a mobility limitation score ≥ 2.09, or a verbal and emotional expression limitation score ≥ 0.62, as these are indicative of chronic temporomandibular disorders. Furthermore, participants were only included if they reported a complete absence of orofacial pain during daily activities, such as eating, talking, or yawning. This screening process was specifically designed to exclude individuals with underlying clinical pathologies, such as muscle spasm, acute inflammatory conditions (e.g., synovitis), or joint hypomobility. By prioritizing functional integrity and the absence of pain, this approach ensured that observed variations in mandibular kinematics represented baseline biomechanical characteristics rather than pathology-driven compensatory patterns.

### Postural assessment

Postural measurements were conducted using the Posture Analyzing and Virtual Reconstruction (PAViR; Moti Physio Pro; MG Solutions, Seoul, Republic of Korea) system. The PAViR system demonstrated an intra-rater reliability ranging from moderate to good (intraclass correlation coefficient [ICC] = 0.69–0.84) and validity ranging from fair to good (r = 0.32–0.79) when compared to EOS (Biospace Med, Paris, France) imaging parameters [20, 21], according to the interpretive thresholds proposed by Portney and Watkins [22]. The PAViR system utilizes background subtraction techniques to extract body silhouettes from depth images, then applies machine learning algorithms, including support vector machines and Superpixel Linear Iterative Clustering, to segment and analyze body posture [20, 21]. To further improve landmark detection accuracy, participants wore form-fitting experimental clothing during assessment. Postural measurements were captured from four views (anterior, lateral, posterior, and Adam’s position), with three repetitions per view. The mean values from these repetitions were used for analysis.

**Fig. 1:**
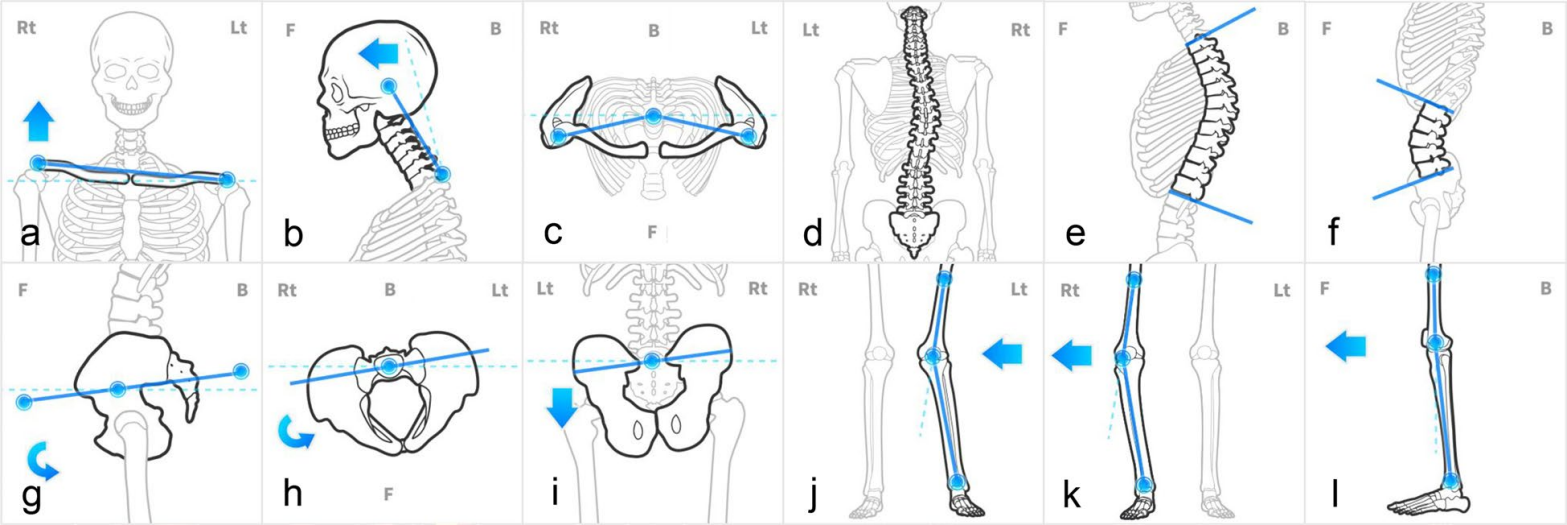
Representative postural analysis parameters. **a** Shoulder height difference; **b** Head posture; **c** Rounded shoulder; **d** Spinal lateral deviation; **e** Thoracic kyphosis; **f** Lumbar lordosis; **g** Pelvic tilt; **h** Pelvic axial rotation; **i** Pelvic obliquity; **j** Left hip-knee-ankle; **k** Right hip-knee-ankle; **l** Knee flexion angle. Image adapted from MotiPhysio with permission

Postural variables were categorized into five regions to ensure a comprehensive assessment of the kinetic chain: head, shoulder, spine, pelvis, and lower extremity. Twelve variables were analyzed: head posture, shoulder height difference, rounded shoulder, thoracic kyphosis, lumbar lordosis, spinal lateral deviation (representing the global lateral curvature of the spine), three pelvic parameters (pelvic tilt, rotation, and obliquity), left and right hip-knee-ankle angles, and knee flexion angle. Notably, these postural variables represent functional angles derived from body surface topography, which are distinct from structural measurements obtained via radiographic imaging. The specific calculation methods and figures for each variable are presented in Table 1 and Fig. 1.

**Table 1:** Posture evaluation criteria

Values	Criteria	Method of calculation
Shoulder height difference	0°	The angle of the point connecting the left acromion and the right acromion
Head posture	11°	The angle formed by the line connecting the spinous process of C7 and the central point of the skull
Rounded shoulder	0°	The angle formed by the lines connecting the lateral ends of both clavicles and the spinous process of C7
Thoracic kyphosis	36°	The average VCM^a^ angle value calculated by accumulating segmental values from T1 to T12 in groups of four vertebrae (e.g., T1–T7, T2–T8, T3–T9)
Lumbar lordosis	35°	The average VCM^a^ angle value calculated by accumulating segmental values from L1 to L5 in groups of three vertebrae (e.g., L1–L3, L2–L4, L3–L5)
Spinal lateral deviation	0°	The average VCM^a^ angle value calculated by accumulating segmental values from T1 to L5 in groups of seven vertebrae (e.g., T1-T7, T2-T8, T3-T9)
Pelvic tilt	0°	The angle of inclination relative to the horizontal line, derived from an AI analysis of body surface topography across four views (anterior, lateral, posterior, and Adam’s)
Pelvic axial rotation	0°	The angle of axial rotation derived from an AI analysis of body surface topography across four views (anterior, lateral, posterior, and Adam’s)
Pelvic obliquity	0°	The angle of lateral inclination (height difference) derived from an AI analysis of body surface topography across four views (anterior, lateral, posterior, and Adam’s)
Left hip-knee-ankle	0°	180°—(the angle formed by three points: Lt. DMF^b^, Lt. Patella, and Lt. Talus)
Right hip-knee-ankle	0°	180°—(the angle formed by three points: Rt. DMF^b^, Rt. Patella, and Rt. Talus)
Knee flexion angle	0°	180°—(Femur head – patella – talus angle)

^a^VCM, Vertebral centroid measurement angle; ^b^DMF, Distal metaphysis of femur

Note: The measurement criteria and definitions for all postural variables were adapted from the MotiPhysio system protocol (MotiPhysio, Seoul, Korea)

### Mandibular movement assessment

Following postural assessment, MMO and MLD were each recorded three times using an iPhone 14 Pro camera (Apple Inc., Cupertino, CA, USA), and the average of the three measurements was used for analysis. While 3D motion analysis systems are considered the gold standard, video-based photogrammetry using high-resolution cameras and Kinovea software (version 0.8.27) has demonstrated excellent reliability and accuracy for linear measurements when validated against reference standards [23]. This method provides a pragmatic and scientifically valid approach for quantifying mandibular deviations in a clinical context.

To ensure the measurement reliability of this digital approach, the intra-rater reliability was calculated. For MLD, the intraclass correlation coefficient was 0.858 (95% confidence interval [CI] = 0.826–0.886), indicating good to excellent reliability, with an internal consistency of 0.948 (Cronbach’s alpha). For MMO distance, the intraclass correlation coefficient was 0.922 (95% CI = 0.896–0.943), also demonstrating excellent reliability, with a Cronbach’s alpha of 0.973.

**Fig. 2:**
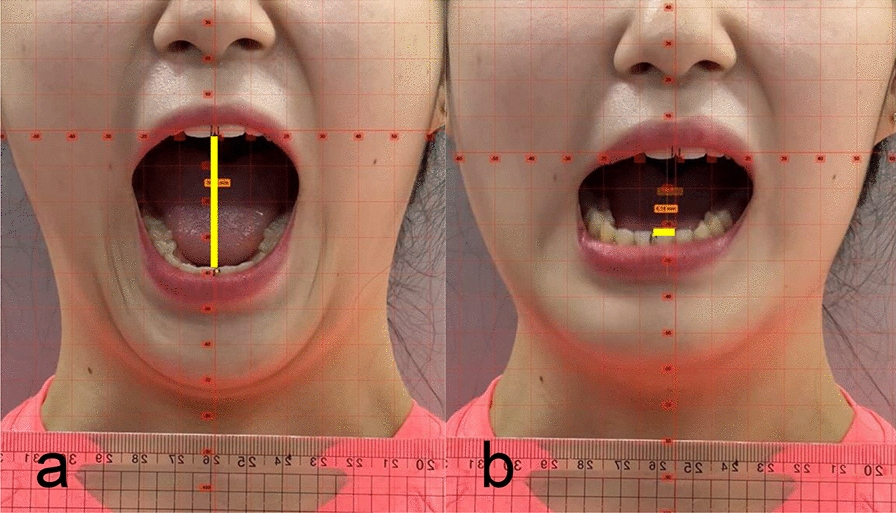
Video-based mandibular movement analysis using Kinovea software. **a** Measurement of maximal mouth opening (MMO), **b** Measurement of mandibular lateral deviation (MLD). Note the calibration ruler visible adjacent to the participant’s face, which was used to set the scale for precise digital measurement

The measurement protocols were adapted from Agerberg [24]; however, this study employed digital video analysis to enhance precision. Participants were seated on a backless chair with their backs and heads lightly touching a wall to standardize head position. A calibration ruler fixed to a wire was positioned close to the face of the participant to ensure accurate scaling. Participants performed MMO and closing movements synchronized to a metronome set at 80 beats per minute. Following three practice trials to establish rhythm familiarity, recordings were captured. A reference baseline was established at the intersection of the maxillary midline and mandibular incisors during occlusion. MMO was measured as the vertical distance between the incisal edges (Fig. 2a), while MLD was defined as the horizontal deviation from the maxillary midline during the complete mouth opening and closing cycle (Fig. 2b).

For statistical analysis, MLD was treated as a signed continuous variable, with positive values ( +) denoting leftward deviation and negative values (−) indicating rightward deviation. In cases where participants demonstrated bilateral deviation during the entire process of opening and closing the mouth, the side with the larger absolute magnitude of lateral deviation was selected to represent the maximal functional asymmetry. Additionally, a subgroup (MMLD > 2 mm) was defined as participants exhibiting a maximal MLD exceeding the clinically relevant threshold of 2 mm, regardless of direction. This threshold was established based on the Diagnostic Criteria for Temporomandibular Disorders [16], which considers deviations within 2 mm to be physiologically acceptable. Thus, a cutoff of 2 mm was used to distinguish minimal variations from more clinically relevant lateral deviations.

### Statistical analysis

Sample size was estimated using G*Power version 3.1 (Franz Faul, University of Kiel, Germany), with an alpha level of 0.05, statistical power of 0.80, and a medium effect size (*f*^2^) of 0.15. A total of 110 participants were recruited. After excluding two statistical outliers identified during data screening, data from 108 participants were used for the final analysis. Although the final sample size was slightly adjusted, a post-hoc power analysis was conducted to confirm the statistical robustness of the study. Based on the observed effect size (*f*^2^ $\approx$ 0.24, derived from the adjusted *R*^2^ of 0.193) and the final sample size of 108 with 12 predictors, the achieved statistical power (1—β) was 0.92, indicating that the sample size was highly sufficient to detect significant associations.

Data normality was evaluated using the Shapiro–Wilk test. As certain variables, including MLD and spinal lateral deviation, deviated from a normal distribution (*p* < 0.05), Spearman’s rank correlation analysis was employed to examine the relationships between variables.

To identify the key postural factors associated with MLD, stepwise multiple linear regression analysis was conducted using MLD as the dependent variable and the 12 postural variables as independent variables. Given the exploratory objective of identifying potential postural correlates among multiple variables, stepwise regression was deemed appropriate. To mitigate Type I error risk and overfitting associated with variable selection, models were evaluated using adjusted *R*^2^ values rather than *p*-values alone, and multicollinearity was rigorously assessed using variance inflation factors (VIF < 5). In all models, VIF values ranged from 1.0 to 1.5, indicating no significant multicollinearity issues. All statistical analyses were performed using SPSS Statistics version 28.0.0.0 (IBM Corp., Armonk, NY, USA), with significance set at *p* < 0.05.

Post-regression diagnostics were performed to validate the model assumptions. Scatterplots of standardized residuals versus predicted values confirmed homoscedasticity, and normal probability (P-P) plots of standardized residuals indicated no major deviations from normality.

## Results

### Participant characteristics

Among the 110 recruited participants, 2 were excluded as statistical outliers (standardized residuals > 2.5). The final analysis included 108 healthy young adults (mean age: 24.28 ± 4.11 years; mean height: 168.45 ± 8.08 cm; mean weight: 68.29 ± 15.38 kg; mean body mass index: 23.86 ± 4.10 kg/m^2^). The detailed clinical and postural characteristics of the participants, including mandibular movement ranges and distributions, are summarized in Table 2.

**Table 2:** General Characteristics (*N* = 108)

	Mean	SD
Age	24.36	4.11
Height (cm)	168.65	8.00
Weight (kg)	68.72	15.18
BMI	23.97	4.06
MLD (mm)	0.98	2.94
MLD_Lt (mm)	1.95	1.73
MLD_Rt (mm)	1.31	1.37
MLD_Total (mm)	3.25	1.74
MMO (mm)	38.39	7.00
Shoulder height difference (°)	1.42	1.58
Head posture (°)	17.57	4.92
Rounded shoulder (°)	13.79	5.42
Spinal lateral deviation (°)	5.33	2.33
Thoracic kyphosis (°)	37.14	6.42
Lumbar lordosis (°)	35.11	5.85
Pelvic tilt (°)	0.01	2.90
Pelvic rotation (°)	1.28	2.21
Pelvic obliquity (°)	−0.37	0.72
Left hip-knee-ankle (°)	1.46	2.72
Right hip-knee-ankle (°)	1.19	2.26
Knee flexion angle (°)	1.74	3.58

SD, Standard deviation; BMI, Body mass index; MLD, Mandibular lateral deviation; MLD_Lt, Mandibular lateral deviation to the left; MLD_Rt, Mandibular lateral deviation to the right; MLD_Total, Mandibular lateral deviation total distance; MMO, Maximal mouth opening distance

### Correlation analysis

Although the knee flexion angle exhibited a statistically significant moderate negative correlation with MLD (ρ = −0.407, *p* < 0.001, Table 3), head posture demonstrated no significant correlation with any of the stomatognathic variables (*p* > 0.05 for all). Notably, the association with lower limb alignment remained robust in the MMLD > 2 subgroup, where the correlation coefficient for knee flexion angle exhibited a slight increase (ρ = −0.419, *p* < 0.001) compared to that in the total sample (ρ = −0.407). Comprehensive correlation coefficients for all 12 postural variables are presented in Table 3.

**Table 3:** Spearman correlation coefficients between the posture and MLD-related variables

Variable($^\circ$\mm)	MLD	MMLD > 2	MMO
Shoulder height difference	− 0.028	− 0.083	0.035
Head posture	0.102	0.083	0.171
Rounded shoulder	− 0.114	− 0.148	− 0.161
Thoracic kyphosis	0.132	0.146	− 0.022
Lumbar lordosis	− 0.025	0.012	− 0.074
Spine lateral deviation	− 0.167	− 0.137	− 0.057
Pelvic tilt	− 0.013	− 0.006	0.038
Pelvic rotation	− 0.104	− 0.120	0.019
Pelvic obliquity	0.001	− 0.099	0.020
Left hip-knee-ankle angle	0.168	0.207	− 0.078
Right hip-knee-ankle angle	0.054	0.115	− 0.110
Knee flexion angle	− 0.407**	− 0.419**	− 0.001

$$*p<0.05$$,$$**p<0.01$$

Values are presented as correlation coefficients

MLD, Mandibular lateral deviation; MMLD > 2, Maximal mandibular lateral deviation > 2 mm regardless of direction; MMO, Maximal mouth opening;

### Regression analysis

Consistent with the correlation findings, the stepwise multiple regression analysis identified knee flexion angle as the primary factor of MLD, with spinal lateral deviation also being included in the final model (Table 4). The final model explained approximately 19.3% of the variance (Adjusted *R*^2^ = 0.193, *p* < 0.001), with the knee flexion angle (β = −0.415, *B* = −0.341, *p* < 0.001) and spinal lateral deviation (β = −0.180, *B* = −0.227, *p* < 0.05) demonstrating significant associations. This trend was even more pronounced in the MMLD > 2 subgroup, where the knee flexion angle emerged as the sole significant factor, explaining 18.1% of the variance (Adjusted *R*^2^ = 0.181, *p* < 0.001). Regarding MMO, rounded shoulder was identified as the only significant factor, albeit the model exhibited limited explanatory power (Adjusted *R*^2^ = 0.028, *p* < 0.05).

**Table 4:** Results of the stepwise multiple regression analysis of the postural predictors for MLD variables and MMO

Dependent variables (mm)	Independent variables ($^\circ )$	Unstandardized coefficients		Standardized coefficients	*t*	*F*	Adj.*R*^2^	DW	VIF
		*B*	SE	β					
MLD	(Constant)	2.781	0.646		4.302**	13.790**	0.193	1.792	
	KFA	− 0.341	0.071	− 0.415	− 4.775**				1.001
	SLD	− 0.227	0.110	− 0.180	− 2.068*				1.001
MMLD > 2	(Constant)	1.769	0.387		4.576**	1.894**	0.181	1.714	
	KFA	− 0.408	0.099	− 0.438	− 4.110**				1.000
MMO	(Constant)	41.816	1.822		22.948**	4.066*	0.028	1.837	
	RS	− 0.248	0.123	− 0.192	− 2.016*				1.000

MLD signs denote direction (+ : left; −: right)

SE, Standard error; Adj.*R*^2^, Adjusted coefficient of determination; DW, Durbin-Watson statistic; VIF, Variance inflation factor; MLD, Mandibular lateral deviation (mm); MMLD > 2, Maximal mandibular lateral deviation > 2 mm regardless of direction; MMO, Maximal mouth opening; RS, Rounded shoulder; SLD, Spine lateral deviation; KFA, Knee flexion angle

$$*p<0.05$$,$$**p<0.01$$

Analysis of postural variables as dependent outcomes revealed substantial bidirectional interrelationships (Table 5). Notably, the models predicting head posture and rounded shoulder using MMO yielded higher explanatory power (Adjusted *R*^2^ = 0.419 and 0.347, respectively) than the MLD model. Additionally, MLD emerged as a significant factor for knee flexion angle (β = -0.410, *p* < 0.001) in a robust model (Adjusted *R*^2^ = 0.257).

**Table 5:** Results of the stepwise multiple regression analysis for posture variables

Dependent variables ($^\circ )$	Independent variables	Unstandardized coefficients		Standardized coefficients	*t*	*F*	Adj.*R*^2^	DW	VIF
		*B*	SE	β					
HP	Constant	− 3.559	3.339		− 1.066	16.422**	0.419	2.164	
	RS ($^\circ )$	0.518	0.069	0.571	7.546**				1.056
	TK ($^\circ )$	0.215	0.057	0.281	3.775**				1.021
	LHKA ($^\circ )$	0.402	0.136	0.222	2.955**				1.042
	MMO (mm)	0.140	0.053	0.200	2.643**				1.054
	PR ($^\circ )$	0.290	0.125	0.171	2.310*				1.007
RS	Constant	10.380	2.755		3.767**	19.942**	0.347	1.415	
	HP ($^\circ )$	0.622	0.087	0.564	7.173**				1.014
	MMO (mm)	− 0.182	0.061	− 0.235	− 2.998**				1.010
	LHKA ($^\circ )$	− 0.357	0.157	− 0.179	− 2.272*				1.017
SLD	Constant	5.479	0.229		23.946**	4.504*	0.061	1.652	
	PT ($^\circ )$	− 0.166	0.075	− 0.207	− 2.212*				1.000
	MLD (mm)	− 0.153	0.074	− 0.193	− 2.061*				1.000
KFA	Constant	2.857	0.358		7.986**	13.332**	0.257	1.568	
	MLD (mm)	− 0.499	0.102	− 0.410	− 4.920**				1.002
	PR ($^\circ )$	− 0.486	0.138	− 0.300	− 3.530**				1.042
	PT ($^\circ )$	− 0.227	0.105	− 0.184	− 2.161*				1.041

$$*p<0.05$$, $$**p<0.01$$

MLD signs denote direction (+ : left; -: right)

SE, Standard error; Adj.*R*^2^, Adjusted coefficient of determination; DW, Durbin-Watson statistic; VIF, Variance inflation factor; MLD, Mandibular lateral deviation; MMO, Maximal mouth opening; HP, Head posture; RS, Rounded shoulder; TK, Thoracic kyphosis; SLD, Spine lateral deviation; PT, Pelvic tilt; PR, Pelvic rotation; LHKA, Left hip-knee-ankle angle; KFA, Knee flexion angle

Finally, to evaluate the robustness of the regression models, we performed a sensitivity analysis incorporating sex as a dummy variable in the initial models. During the stepwise selection process, sex was excluded as it was not a significant factor (*p* > 0.05) and did not contribute to the explanatory power of the model. Consequently, data were pooled for the final analyses. Collinearity diagnostics revealed no evidence of multicollinearity, with all VIF values ranging from 1.00 to 1.06.

## Discussion

Contrary to established literature emphasizing the association between the temporomandibular region and the craniocervical complex, the most significant finding of the present study is that MLD in healthy young adults is more closely associated with the stability of the lower kinetic chain, specifically the knee flexion angle in the sagittal plane and spinal lateral deviation. This shift suggests that global postural factors may play a more substantial role in mandibular kinematics than previously recognized, reinforcing the clinical relevance of knee flexion angle as a potential postural indicator in asymptomatic populations.

In the present study, knee flexion angle measured in the standing position in the sagittal plane exhibited a negative correlation with MLD variables, an association that was more pronounced in participants whose maximal MLD exceeded 2 mm. Furthermore, regression analyses identified knee flexion angle as a significant factor associated with both general MLD and the maximal MLD exceeding 2 mm subgroup. Notably, this angle was the sole important factor in the maximal MLD exceeding 2 mm in the model. The observed negative correlation suggests that alterations in knee alignment are intrinsically linked to directional deviations of the mandible. Notably, this trend was consistent in both correlation and regression analyses, underscoring the statistical and clinical significance of these findings. In terms of clinical interpretation, the unstandardized coefficient (*B*) suggests that a 1-degree difference in knee flexion corresponds to an estimated 0.341 mm rightward lateral mandibular deviation (*B* = −0.341, β = -0.415). Similarly, a 1-degree difference in spinal lateral deviation is associated with an estimated 0.227 mm rightward lateral deviation (*B* = −0.227, β = −0.180).

The emergence of spinal lateral deviation as a significant factor associated with mandibular deviation supports the view that managing TMDs requires an extensive approach. As emphasized by Miçooğulları et al., clinical consideration should extend beyond the jaw to encompass the entire cranio–cervico–mandibular system and the spinal complex [25, 26]. Our findings provide empirical support for this integrated view, suggesting that spinal alignment is intrinsically linked to mandibular symmetry even in an asymptomatic population. However, spinal lateral deviation was identified as a significant factor only in the regression model, not in the bivariate correlation, suggesting that its role may be less prominent or context-dependent, rather than exhibiting the direct, robust relationship observed for knee flexion angle. This is further evidenced by the comparison of standardized coefficients β, which reveals that knee flexion angle (β = −0.415) exerted a substantially greater relative influence on MLD than spinal lateral deviation (β = −0.180).

While the adjusted *R*^2^ values of 0.19 indicate a modest explained variance, this association is noteworthy given the multifactorial complexity of human posture. Considering the anatomical distance between the stomatognathic system and the lower extremities, the finding that knee alignment accounts for approximately 20% of the variance in mandibular deviation suggests a potential biomechanical interconnection rather than a coincidental finding.

This knee-mandible relationship is structurally grounded in the myofascial continuity theory. Myers [27] and Stecco et al. [28] described how the deep fascia of the lower limb connects continuously with the thoracolumbar fascia, extending upward to the cervical region. This anatomical continuity appears to be consistent with previous clinical studies [29–31] demonstrating that myofascial interventions in the lower limbs influence the stomatognathic system. Specifically, Espejo-Antúnez et al. [29] reported that hamstring stretching immediately increased MMO distance and elevated masseter pressure pain thresholds in athletes with TMD. Bretischwerdt et al. [30] observed similar immediate improvements, specifically in healthy participants. Furthermore, Rodríguez-Blanco et al. [31] confirmed that combining hamstring stretching with local craniomandibular techniques improved both mouth opening and lower limb and cervical ranges of motion. The consistency across these findings, involving both clinical and healthy populations, points toward the possibility that the myofascial link between the lower limbs and the stomatognathic system is not exclusive to pathological states but represents a fundamental biomechanical relationship. These experimental results indicate that the significant association involving the knee flexion angle is not merely a reflection of the widespread prevalence of hamstring tightness in modern populations. Specifically, these results, demonstrating that targeting the lower limbs produces immediate changes in the jaw, provide further evidence that this relationship represents a functional biomechanical link rather than a coincidental association.

Beyond structural connectivity, this relationship also has a neurophysiological basis. Proprioceptive inputs from the lower extremities are essential for postural control and are centrally integrated with trigeminal afferents [8, 25, 32]. Furthermore, recent clinical evidence demonstrates that malalignment or sensory disruptions within the cranio–cervico–mandibular system can generate abnormal afferent information that impairs neuromuscular control, leading to increased total body sway and reduced joint position sense. This suggests that the maintenance of mandibular symmetry may be hindered by postural or sensory interference from distant body regions [25, 26]. Proprioceptive feedback from the TMJ and associated muscles has been suggested to be centrally integrated with visual and vestibular inputs, subsequently influencing muscle tone and global posture [33]. This collective integration of multisensory inputs aligns with the characterization of the stomatognathic system as a “dynamic sensorimotor unit” for postural control. Consequently, knee instability or misalignment may be associated with compensatory muscle activation patterns that ascend the kinetic chain, ultimately manifesting as MLD.

These structural and neurological mechanisms can be framed within a broader biomechanical framework of gravity-driven bottom-up postural organization. Posture begins where the body meets the ground: at the feet. As the lower kinetic chain serves as the foundation for global alignment, instability or misalignment in the lower extremities, such as increased knee flexion angle, may initiate an ascending cascade of postural adjustments [34]. In response to this structural instability, the stomatognathic system, situated at the top of the kinetic chain, may act as a fine-tuner for global balance [33]. By adjusting its position (e.g., MLD), the jaw may facilitate compensatory changes in head and trunk orientation to maintain the body’s center of mass within the base of support. This systemic interdependence is further supported by evidence that dysfunction in any part of the kinetic chain, even as distal as the foot, can influence the activation of masticatory muscles [35]. Such findings reinforce the view that managing mandibular issues should not be limited to the jaw itself but must encompass the entire spinal complex and lower extremities as a single functional unit. Consequently, the MLD observed in our study may not be a primary pathology but rather a compensatory manifestation to maintain gaze stability and equilibrium in response to ascending dysfunction from the lower body. Supporting this view, recent clinical trials have demonstrated that therapeutic interventions targeting the myofascial chains can simultaneously improve both mandibular and spinal function, empirically supporting this biomechanical connectivity across distant body regions [25, 31, 36].

However, the influence of this ascending model does not apply uniformly to all mandibular parameters. Notably, in the present study, the factors influencing mouth opening distance differed distinctively from those affecting MLD. Specifically, while mandibular deviation was associated with lower body variables, MMO distance was significantly associated with rounded shoulder, an upper trunk variable. Our finding of a significant association between mouth opening distance and rounded shoulder is supported by prior research demonstrating that patients with TMD exhibit an increased prevalence of protracted shoulders and head-forward posture [37]. The biomechanical mechanism underlying this association can be explained through the postural interdependence between the shoulder girdle and the craniomandibular system [10]. This association may also involve neuromuscular sensitization through the trigeminocervical complex, where afferent inputs from the upper cervical and shoulder regions modulate mandibular motor control [8, 11].

This mechanistic framework helps explain why our findings differed from previous clinical studies. The discrepancies likely reflect both the heterogeneity of participant characteristics and the specific nature of the dependent variables analyzed. First, regarding the dependent variables, our results demonstrated that MMO distance, which is a measure of functional capacity, was significantly associated with rounded shoulders; this is consistent with existing TMD literature focusing on proximal upper trunk alignment. However, when we introduced mandibular deviation, we observed a significant association with the lower kinetic chain. This finding suggests that while the range of mouth opening is primarily influenced by proximal alignment, such as rounded shoulder and neck posture, mandibular deviation is more sensitive to global alignment and the ascending kinetic chain originating from the lower limbs. Although knee flexion angle is measured in the sagittal plane, its influence on overall body symmetry and the compensatory postural shifts along the ascending chain can ultimately manifest as lateral deviation of the mandible. Second, the pain adaptation model further explains why these global associations were particularly evident in our asymptomatic population. In patients with TMD, nociceptive inputs often lead to protective guarding of the neck and shoulders, which may mask the subtle, long-range influences originating from the lower body [38]. In contrast, our healthy participants were free from such pain-mediated constraints, allowing the direct biomechanical influence of the ascending chain—from the lower limbs to the mandible—to emerge without interference [39]. Thus, by focusing on mandibular deviation in a healthy population, we captured a baseline functional connectivity that is often obscured in symptomatic clinical trials.

Despite the distinct regional associations for mouth opening distance and mandibular deviation, our final analysis underscores that these components do not function in isolation. The analysis of postural variables as dependent outcomes (Table 5) further substantiates the bidirectional nature of these postural relationships. Specifically, MMO demonstrated notably high explanatory power in predicting head posture and rounded shoulder (adjusted *R*^2^ = 0.419 and 0.347, respectively), suggesting that the stomatognathic system may serve as a primary determinant of upper body alignment. These observations are consistent with Makofsky’s “Sliding Cranium Theory,” which posits that the cranium “slides” over the cervical spine relative to the mandible [40]. According to this framework, the craniocervical repositioning driven by mandibular function necessitates a descending cascade of postural compensations, ultimately reshaping the alignment of the shoulder girdle and thoracic spine to maintain global equilibrium.

Overall, our findings suggest that the jaw function extends beyond local biomechanics; the jaw can respond to ascending influences from the lower limbs while simultaneously dictating the alignment of the upper trunk. This bidirectional relationship suggests that the stomatognathic system acts as a “dynamic sensorimotor unit,” where alterations in mandibular functional range may necessitate reciprocal adjustments in the upper kinetic chain to optimize visual and vestibular orientation. Therefore, the body functions not as a collection of independent segments, but as an integrated, reciprocal unit where dysfunction at any level (e.g., knee, jaw, or shoulder) can propagate through the kinetic chain.

### Strengths and limitations

The strengths of this study include its relatively large sample size (n = 108) for exploratory postural research, the use of validated 3D posture and video analysis systems, and the simultaneous assessment of 12 global postural variables. Furthermore, by focusing on healthy adults, our study provides valuable normative data that can serve as a baseline for future comparative research involving symptomatic populations.

However, this study has certain limitations. First, the cross-sectional design precludes causal inferences, preventing a definitive conclusion on whether knee posture influences the mandible or vice versa. Second, as the participants were healthy young adults, generalization of our findings to patients with TMD should be cautious. Third, although the video-based analysis used for MLD has demonstrated high reliability and clinical utility, it may lack the depth-sensing precision of 3D motion capture systems, which remain the gold standard for kinematic assessment. Fourth, regarding the postural assessment, the pelvic parameters provided by the PAViR system are derived from an artificial intelligence-based analysis of surface topography rather than specific anatomical landmarks used in radiographic imaging. While these variables provide valuable functional insights into global alignment, they should be interpreted with caution when compared to standard radiographic measurements. Fifth, potential confounding factors, such as handedness and the dominant chewing side, were not strictly controlled. Although a homogeneous group of healthy young adults was recruited to minimize variability, future studies should consider these variables as covariates to further refine predictive models. Sixth, in clinical practice in the Republic of Korea, a diagnosis of TMD typically involves radiographic assessments, such as computed tomography, to evaluate structural integrity. However, to avoid unnecessary radiation exposure in asymptomatic healthy volunteers, this study prioritized ethical considerations and instead utilized a validated screening approach. Nevertheless, TMDs were not formally assessed using standardized diagnostic tools, such as the Diagnostic Criteria for Temporomandibular Disorders. Given that some participants may have exhibited limited mouth opening, which is a cardinal clinical sign of TMDs, the absence of a structured diagnostic protocol represents a methodological limitation and should be considered when interpreting the findings. Finally, given the exploratory nature of this study, strict Bonferroni corrections were not applied to avoid enhancing Type II errors. While this approach theoretically increases the Type I error risk, the consistency of our findings across different statistical methods suggests robustness. However, we acknowledge that the explained variance in this study was modest (adjusted *R*^2^ $\approx$ 0.19), which suggests that other unmeasured factors likely contribute to mandibular deviation. Therefore, future confirmatory studies with larger sample sizes and longitudinal designs are warranted to establish causality and clinical predictive value.

## Conclusion

This study demonstrates a distinct regional association between global posture and mandibular kinematics in healthy young adults. Our findings reveal that while the range of mouth opening is primarily linked to proximal upper trunk alignment, specifically rounded shoulder posture, MLD is significantly associated with the lower kinetic chain, particularly the knee flexion angle. The identification of knee alignment in the sagittal plane as a significant factor in our models underscores a potential biomechanical interconnection between the stomatognathic system and the lower extremities. Within the framework of the pain adaptation model, these findings suggest that the absence of pain-mediated protective mechanisms allows baseline functional interdependence along the ascending kinetic chain to emerge more clearly. However, given the multifactorial complexity of human posture and the cross-sectional nature of this study, these global factors should be interpreted as contributing elements rather than definitive causal determinants.

Clinically, these results support a differentiated assessment approach based on clinical presentation. For patients with limited mouth opening, clinicians should evaluate proximal shoulder and neck alignment. In contrast, for patients presenting with mandibular deviation, a more comprehensive evaluation, including lower limb and knee alignment, is warranted. Future longitudinal research is necessary to further elucidate the underlying causal mechanisms and validate the predictive value of these postural indicators in clinical populations.

## Data Availability

The datasets used and/or analyzed during the current study are available from the corresponding author on reasonable request.

## Abbreviations

TMJ: Temporomandibular joint
MLD: Mandibular lateral deviation
TMDs: Temporomandibular disorder
MMO: Maximal mouth opening
MMLD > 2: Maximal mandibular lateral deviation exceeding 2 mm regardless of direction
Adj.*R*^2^: Adjusted coefficient of determination
